# Spontaneous Subcapsular Renal Hematoma: Strange Case in an Anticoagulated Patient with HWMH after Aortic and Iliac Endovascular Stenting Procedure

**DOI:** 10.1155/2016/2573476

**Published:** 2016-08-08

**Authors:** Michele Greco, Salvatore Butticè, Filippo Benedetto, Francesco Spinelli, Olivier Traxer, Tzevat Tefik, Rosa Pappalardo, Carlo Magno

**Affiliations:** ^1^Cardiovascular and Thoracic Department, Unit of Vascular Surgery, Policlinico G. Martino Hospital, University of Messina, 98124 Messina, Italy; ^2^Department of Human Pathology, Unit of Urology, Policlinico G. Martino Hospital, University of Messina, 98124 Messina, Italy; ^3^Unit of Vascular Surgery, Campus BioMedico University, 00128 Rome, Italy; ^4^Pierre & Marie Curie University, Tenon University Hospital, 75020 Paris, France

## Abstract

Spontaneous subcapsular renal hematoma is a rare condition in clinical practice. It is caused by renal cysts, benign and malignant renal tumors, vascular lesions, and antiplatelet or anticoagulant therapy. In this paper we report an unusual case of rupture of a renal cyst of a 66-year-old male patient during an aortic and iliac endovascular procedure for a massive calcified atheroma above the iliac bifurcation. We suspected that the bolus of high weight molecular heparin given during the procedure caused the rupture of the cyst. According to the literature, this is the first case of renal cyst rupture during an endovascular aortic procedure after administering a high weight molecular heparin bolus.

## 1. Introduction 

Spontaneous subcapsular renal hematoma (SPH) is a diagnostic dilemma and a rare condition in clinical practice. There are different etiologies that include benign and malignant renal tumors, renal infections, and vascular lesions. Renal cysts, blood dyscrasias, or anticoagulant and antiplatelet therapy are some of the less common causes. The presentation of symptoms and clinical signs depends on the degree and duration of bleeding and may therefore vary significantly. We present SPH in an anticoagulated patient after administration of high weight molecular heparin (HWMH) bolus, during an aortic and iliac endovascular stenting procedure [1–3].

## 2. Case Presentation 

A 66-year-old male patient was presented to us with severe intermittent claudication at the left lower limb (<50 m). This was caused by a massive calcified atheroma above the iliac bifurcation, documented by a preoperative CT scan. The CT scan also showed an 18 mm renal cyst at the third medium of the right kidney. The patient underwent endovascular infrarenal aortic stenting (CP Stent*™*, NuMed Inc.) and 9 mm iliac bilateral kissing stenting (E·LUMINEXX®, Bard Peripheral Vascular) with good results and his symptoms disappeared. During the endovascular procedure, it is common to give a bolus of HMWH and the patient was given a bolus of 2000 UI, according to his weight. Twelve hours after the procedure, the patient complained of abdominal pain on his right side, mostly at the hypochondrium; this subsided after taking pain medication (ketorolac trometamina iv).

**Figure 1:**
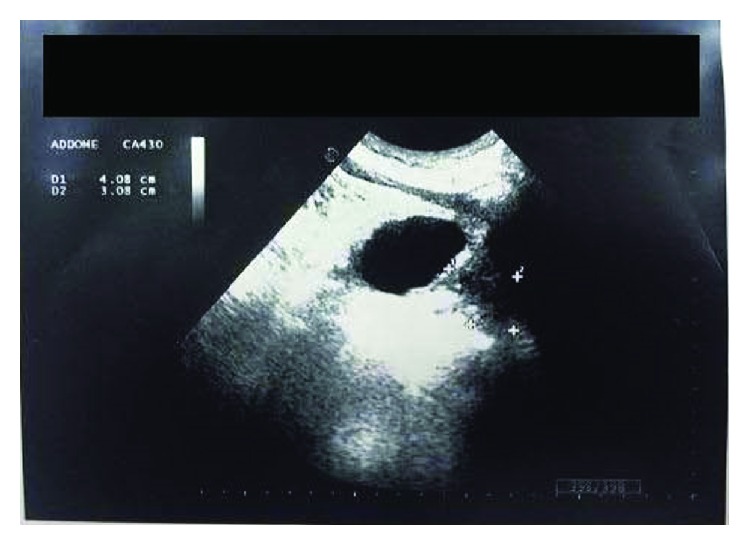
Ultrasound aspects of the renal hematoma.

**Figure 2:**
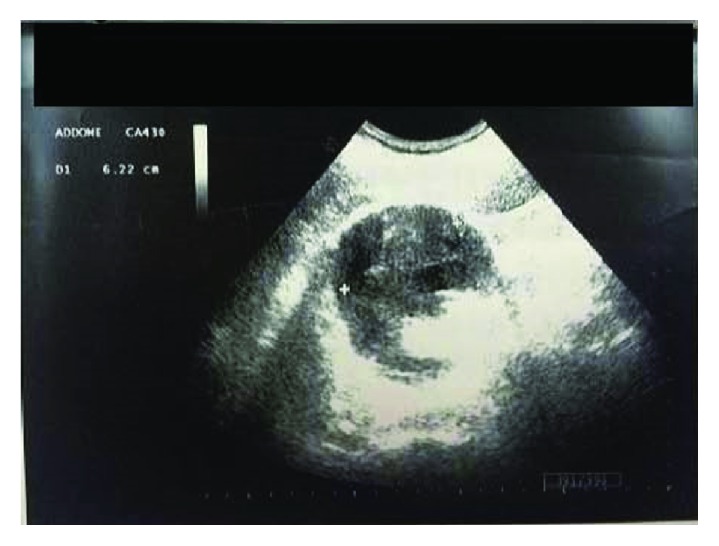
Ultrasound aspects of the renal hematoma.

We suspected a biliary colic because of the presence of a 14 mm gallstone and a positive Murphy's sign. Thus, the patient underwent an abdominal US that found “no dilatations of the bile ducts and the presence, in the right kidney, of a solid formation, probably due to hematoma” (Figures 1 and 2).

**Figure 3:**
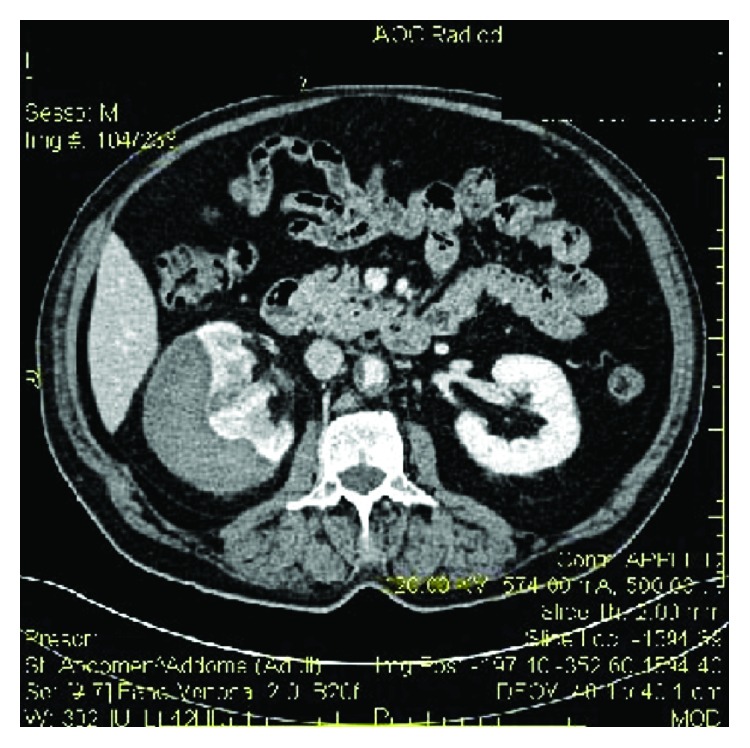
CT aspect of the hematoma.

**Figure 4:**
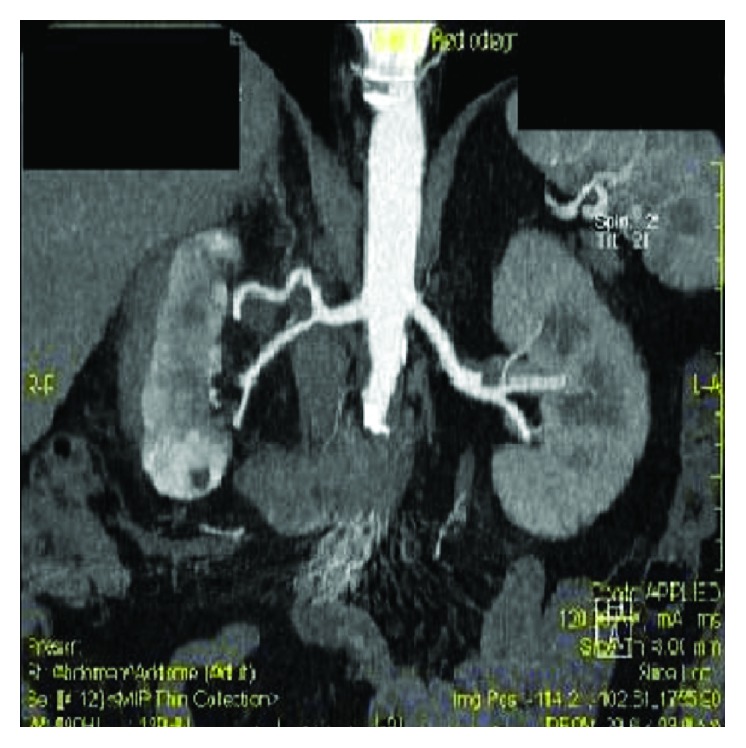
3D CT reconstruction.

A CT scan confirmed the suspicion (Figures 3 and 4). An extended perirenal subcapsular hematoma was detected in the right kidney with a maximum axial thickness of approximately 46 mm, with contrast enhancement in the late stages of the study. It was associated with a full-thickness rupture in the renal parenchyma, with a maximum size of more than 4 cm, that largely covered the middle third and lower pole of the right kidney. The first instance can be attributed to a plurifocal renal breakage. The laboratory examinations revealed that haemoglobin levels, renal function, coagulation profile, and urine test were in the normal ranges.

**Figure 5:**
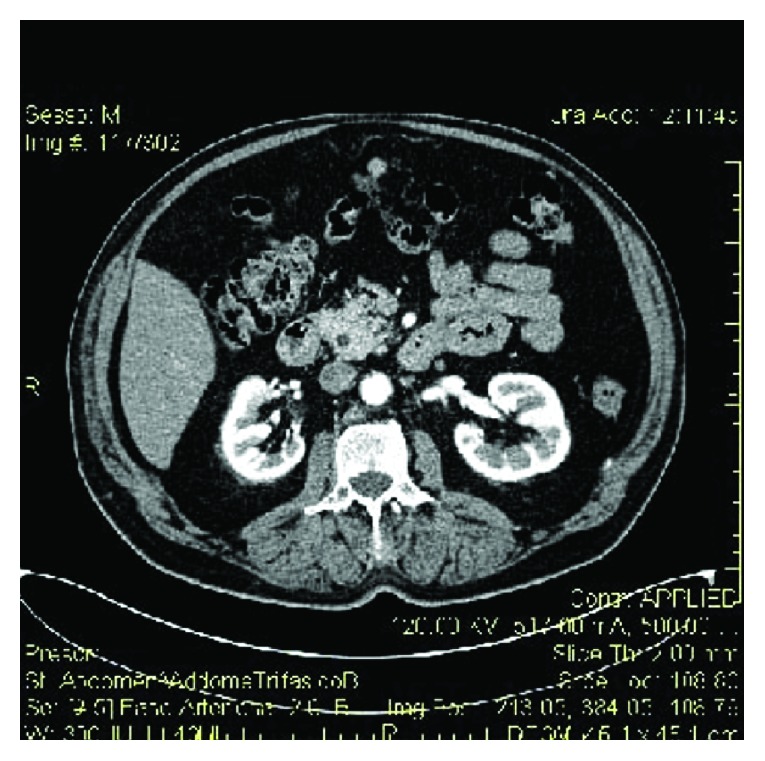
Follow-up CT arterial phase.

**Figure 6:**
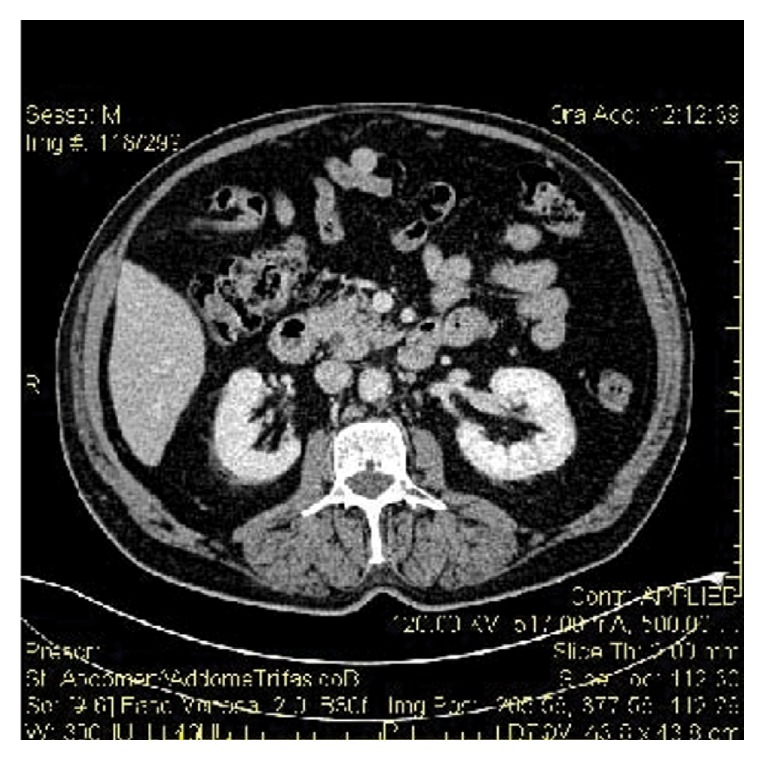
Follow-up CT venous phase.

At this point we opted for a conservative approach. We followed the patient up with blood tests and an ultrasound each day and symptoms diminished within two days. The patient was discharged after seven days. We performed a CT after 3 months that showed a complete resolution of the subcapsular hematoma and reduced renal laceration, with remaining hypodense foci that indicated interruption from cortical to the medullary part of the kidney: this scenario was compatible with a significant regression of the SPH (Figures 5 and 6).

## 3. Discussion 

Spontaneous renal hematoma was initially reported by Bonet in 1679. It was later described by Wunderlich in 1856 [4, 5].

It is characterized by acute onset of spontaneous renal bleeding confined to the subcapsular and perirenal spaces [6]. The syndrome is commonly associated with various pathological conditions such as neoplasm and vascular disease. Accounting for ~60–65% of all cases, renal neoplasms are the most common cause of SPH. Angiomyolipoma is the most common benign neoplasm responsible for SPH, while renal cell carcinoma is the most common malignant neoplasm [7].

Other less common causes are vascular diseases 17%, idiopathic cases 6.7%, and infections 2,4% [8]. SPH may be present with “Lenk's triad” which consists of lumboabdominal pain, palpable mass, and a general deterioration with hypovolemic shock [9].

It may mimic acute abdominal conditions like acute appendicitis, a perforated viscus, or a dissecting aneurysm [10]. The diagnosis is often complex. The first step is an ultrasound, which often does not identify renal masses or small abscesses [11]. The findings of the ultrasonography have to be confirmed by a CT scan. CT scans give valuable information regarding the cause of the hematoma, as they are more sensitive than ultrasounds.

Magnetic Resonance Imaging (MRI) is an alternative if a CT scan fails to identify an active bleeding source [7].

When it comes to the treatment of SPH there are different schools of thought. Bosniak believes that using a CT scan with contrast sections of 5 mm is sufficient to make an accurate diagnosis and that explorative laparotomy is not necessary [12].

If the etiology cannot be determined upon primary examination, a follow-up CT scan should be performed at three-month intervals until the hematoma resolves itself and a definite diagnosis can be made [11, 13].

In contrast, Kendall et al. recommend a nephrectomy when there is not a clear diagnosis and when the contralateral kidney is functionally normal, due to the high incidence of small tumors associated with this condition [14].

Others prefer renal arteriography with embolization, both for diagnosis and therapeutic purposes. Although there are not yet evidence-based guidelines to favor either approach, transarterial embolization has become the choice of initial treatment for renal hemorrhage, irrespective of causes [15, 16]. In our case, we gave a bolus of HWMH because the patient had no risk factors for SPH and the CT scans showed no renal neoplasms.

In 2006 Ferrando et al. reported a spontaneous subcapsular hematoma in an anticoagulated patient with oral anticoagulation but this appears to be the only case after taking HWMH during an aortic endovascular procedure [17].

After the hematoma diagnosis and the discharge of the patient, due to the high risk of thromboembolism, the administration of a single dose per day of low weight molecular heparin (enoxaparin 0.2 mL) was continued. There was no evidence of problems related to the administration of LWMH. After 30 days, we suspended the LWHM administration and started the patient on an antiplatelet drug. It is obvious that a close partnership between vascular surgeons and urologists is essential to achieving the best therapy. For these reasons, we conclude that, in patients with high cardiovascular or thromboembolic risk, continuing therapy with LWMH does not seem to interfere with the trend of SPH.
